# *Lactobacillus salivarius* UCC118™ Dampens Inflammation and Promotes Microbiota Recovery to Provide Therapeutic Benefit in a DSS-Induced Colitis Model

**DOI:** 10.3390/microorganisms10071383

**Published:** 2022-07-09

**Authors:** Namrata Iyer, Michelle A. Williams, Amy A. O’Callaghan, Elaine Dempsey, Raul Cabrera-Rubio, Mathilde Raverdeau, Fiona Crispie, Paul D. Cotter, Sinéad C. Corr

**Affiliations:** 1 School of Genetics and Microbiology, Moyne Institute for Preventative Medicine, Trinity College Dublin, D02 R590 Dublin, Ireland; iyern@tcd.ie (N.I.); william5@tcd.ie (M.A.W.); ocallaa4@tcd.ie (A.A.O.); dempseel@tcd.ie (E.D.); 2 APC Microbiome Ireland, Co., T12 K8AF Cork, Ireland; raul.cabrerarubio@teagasc.ie (R.C.-R.); fiona.crispie@teagasc.ie (F.C.); paul.cotter@teagasc.ie (P.D.C.); 3 School of Biochemistry and Immunology, Trinity Biomedical Sciences Institute, Trinity College Dublin, D02 R590 Dublin, Ireland; raverdem@tcd.ie; 4 Teagasc Food Research Centre, Moorepark, P61 C996 Cork, Ireland

**Keywords:** probiotic, microbiota, inflammation, colitis

## Abstract

The use of probiotics such as *Lactobacillus* and *Bifidobacterium* spp. as a therapeutic against inflammatory bowel disease (IBD) is of significant interest. *Lactobacillus salivarus* strain UCC118^TM^ is a commensal that has been shown to possess probiotic properties in vitro and anti-infective properties in vivo. However, the usefulness of UCC118 ^TM^ as a therapeutic against colitis remains unclear. This study investigates the probiotic potential of *Lactobacillus salivarius*, UCC118™ in a mouse model of colitis. DSS-induced colitis was coupled with pre-treatment or post-treatment with UCC118^TM^ by daily oral gavage. In the pre-treatment model of colitis, UCC118^TM^ reduced the severity of the disease in the early stages. Improvement in disease severity was coupled with an upregulation of tissue IL-10 levels and increased expression of macrophage M2 markers. This anti-inflammatory activity of UCC118^TM^ was further confirmed in vitro, using a model of LPS-treated bone marrow-derived macrophages. Taken together, these results suggest that UCC118^TM^ may promote the resolution of inflammation. This was supported in a mouse model of established DSS-induced colitis whereby UCC118^TM^ treatment accelerated recovery, as evidenced by weight, stool, histological markers and the recovery of microbiome-associated dysbiosis with an increased abundance of beneficial commensal species. These results demonstrate the potential of *Lactobacillus salivarius* UCC118^TM^ as a probiotic-based therapeutic strategy to promote health through the upregulation of anti-inflammatory IL-10 and protect against dysbiosis during IBD.

## 1. Introduction

Inflammatory bowel disease (IBD) is a group of relapsing-remitting disorders, including Crohn’s disease and ulcerative colitis, that is typically a lifelong chronic illness [1]. IBD has a mixed and poorly-understood aetiology with worldwide incidence on the rise [2]. It is widely accepted that IBD is the result of a combination of genetic predisposition and environmental factors associated with a dysfunction in gut-barrier function, microbial dysbiosis and over-activation of immune cells. There is much interest in exploring the gut microbiota to reveal IBD therapies, particularly the use of probiotic bacteria [3].

*Lactobacillus* and *Bifidobacterium* spp. have emerged as promising candidates to be used as probiotics for the treatment and prevention of intestinal disease [4,5]. A crucial goal of research within the field is to determine the precise mechanisms governing the mode of action of probiotics. Emerging research has identified underlying mechanisms that include immunomodulation, production of short-chain fatty acids and the direct inhibition of pathogens by the production of antibacterial substances [6,7,8]. *Lactobacillus salivarius* UCC118™ is a commensal that was first isolated from the gut of an adult undergoing reconstructive surgery [9,10]. The probiotic potential of *Lactobacillus salivarius* UCC118™ in the prevention of intestinal infection has been demonstrated. It exerts direct anti-bacterial activity through the production of a bacteriocin that selectively inhibits the foodborne pathogen *Listeria monocytogenes* [11]. However, to date, there has been limited work carried out to determine any potential for this strain in the treatment of intestinal inflammation, in particular IBD [10,12,13].

Here, we evaluate the probiotic potential of *Lactobacillus salivarius* UCC118™ in a mouse model of DSS-induced colitis. Using a pre-treatment model, we show that oral administration of UCC118™ before induction of DSS-induced colitis serves to reduce overall disease activity. UCC118™ treatment was associated with an increase in the levels of the anti-inflammatory cytokine interleukin-10 (IL-10). Using bone marrow-derived macrophages, we demonstrate that treatment with UCC118™ strain upregulates the expression of M2 macrophage markers and IL-10 secretion. Further, we observed that UCC118™ strain was highly effective at promoting recovery from DSS-colitis with a concomitant shift in the gut microbiota profile favouring the abundance of health-associated bacteria. Our results suggest that UCC118™ can dampen intestinal inflammation and reduce microbial dysbiosis to provide therapeutic benefit in a mouse model of intestinal inflammation.

## 2. Materials and Methods

### 2.1. Mice

Animal studies were performed in C57BL/6JOlaHsd mice. Mice were maintained in ventilated cages at 21 ± 1 °C, humidity 50 ± 10%, with a 12 h light/12 h dark light cycle under specific pathogen-free conditions, in line with Irish and European Union regulations. Food and water were monitored and available *ad libitum* throughout the experiments. All experiments were subject to ethical approval by Trinity College Dublin’s Animal Research Ethics Committee and were carried out in accordance with the Irish Health Products Regulatory Authority, the competent authority responsible for the implementation of Directive 2010/63/EU on the protection of animals used for scientific purposes in accordance with the requirements of the S.I No 543 of 2012.

### 2.2. Culture and Administration of Lactobacillus salivarius UCC118™

The *Lactobacillus salivarius* UCC118™ strain was gifted by PrecisionBiotics Group Ltd. and Prof. Paul O’Toole and was grown in deMan, Rogosa and Sharpe (MRS, Oxoid) anaerobically at 37 °C, in an Oxoid 2.5 L chamber for 18–24 h or until stationary phase was reached (OD_600 nm_ 1.7). UCC118™ was administered to mice by oral gavage (1 × 10^9^ CFU/mouse in a 100µL volume sterile phosphate buffered saline (PBS)) every day for 1 week prior to and during induction of DSS-colitis. Untreated mice received sterile PBS (100 µL) by oral gavage. To assess the ability of UCC118™ to promote recovery from DSS-colitis, mice were administered either sterile PBS or UCC118™ (1 × 10^9^ CFU/mouse in 100 µL volume) daily by gavage during the recovery period.

### 2.3. DSS-Induced Colitis

Colitis was induced in age and sex-matched mice by administration of 2.5% DSS (36–50 kDa, MP Biomedicals, CA, USA) *ad libitum* via drinking water for 6 days unless otherwise stated. Control mice received normal drinking water. Mice were scored every day for disease activity including diarrhoea, faecal occult blood and body weight. The Disease Activity Index (DAI) was calculated every day for each mouse based upon individual scores (diarrhoea, blood, weight loss). The maximum DAI score was 4 based on an average of a 0–4 scoring system for each parameter: score 0, no weight loss, normal stool and no blood; score 1, 1–3% weight loss; score 2, 3–6% weight loss, loose stool (a loose stool was defined as the formation of a stool that readily becomes paste upon handling) and blood visible in stool; score 3, 6–9% weight loss; and score 4, >9% weight loss, diarrhoea and gross bleeding. At the end of the trial, mice were sacrificed by CO_2_ asphyxiation. Serum was generated following terminal bleed. The colons were removed and colon length measured as an indication of colonic inflammation. Subsequently, distal colon sections were taken and fixed in 10% formalin for histology or snap frozen at −80 °C.

### 2.4. DSS-Induced Colitis Recovery Model

Mice were administered 2.5% DSS for 5 days, followed by the removal of DSS on day 6 and replacement with water to allow a period of recovery over 5 days (Day 6–10). During the recovery period, mice were administered daily either sterile PBS or UCC118™ by oral gavage. Mice were scored every other day for disease activity as above. Mice were sacrificed on the final day of the trial by CO_2_, asphyxiation, with colons removed and length recorded and samples harvested for further analysis.

### 2.5. Histological Analysis of Colon Sections

One centimetre sections were taken from the distal colon of each mouse, fixed in 10% buffered formalin and mounted in paraffin blocks, and subsequently Hemotoxylin and Eosin (H&E) staining was performed. H&E-stained sections were blind-scored to determine the extent of colitic injury as described previously [14]. A combined score of inflammatory cell infiltration and tissue damage was determined as follows: cell infiltration: score 0, occasional inflammatory cells in the lamina propria (LP); 1, increased infiltrate in the LP predominantly at the base of crypts; 2, confluence of inflammatory infiltrate extending into the mucosa; 3, transmural extension of infiltrate. Tissue damage: score 0, no mucosal damage; 1, partial (up to 50%) loss of crypts in large areas; 2, partial to total 50–100% loss of crypts in large areas, epithelium intact; 3, total loss of crypts in large areas and epithelium lost. Sections were imaged on an Olympus BX51 light microscope.

### 2.6. Colonic RNA Extraction and qRT-PCR

Colon sections were weighed and homogenised in RNA lysis buffer (PureLink RNA mini kit, Ambion, Austin, TX, USA) by physical disruption with RNAse-free metal ball bearings in a Qiagen TissueLyser II at 20 Hz for 1.5 min. Following homogenisation, total RNA was extracted as per manufacturer’s instructions (PureLink RNA mini kit, Ambion) and treated with DNase 1 to remove DNA contamination. cDNA was prepared for general qRT-PCR using the Applied Biosystems (AB) High Capacity cDNA synthesis kit. Expression of other gene targets was analysed using SYBR green reagents (PowerUp SYBR green, AB) and represented as fold change in expression relative to housekeeping gene RPS13. Primer sequences are detailed in Table 1.

### 2.7. Colonic Myeloperoxidase (MPO) Assay

Colon sections were weighed and homogenised in ice-cold MPO-buffer 1 (see Supplementary Material) and centrifuged at 4 °C for 10 min at 10,000 g. The pellet was subjected to hypotonic lysis in a solution of sodium chloride and glucose, resuspended in MPO buffer 2 and subjected to freeze–thaw cycles followed by further centrifugation. The resulting supernatant was harvested and diluted 1:2, and MPO enzyme activity was measured colorimetrically using TMB substrate solution (BD Biosciences). Activity was denoted as arbitrary units per 100 mg of tissue.

### 2.8. Caco-2 Cell Culture and Measurement of Transepithelial Electrical Resistance

A human Caco-2 adenocarcinoma cell line was used to model intestinal epithelial cells. Caco-2 cells were maintained at 37 °C plus 5% CO_2_ and 95% relative humidity in complete Dulbecco’s Modified Eagle Medium (DMEM; Gibco) supplemented with 10% foetal calf serum (FCS; Gibco) and 1% penicillin–streptomycin (PS; Sigma, St Louis, MO, USA). For use in experiments, Caco-2 cells were seeded onto 12-well transwell plates (3 µM pore-size polycarbonate membranes, Costar, Corning, New York, NY, USA) at a density of 6 × 10^4^ cells/cm^2^ and allowed to differentiate for 21 days or until transepithelial electrical resistance (TER; Ω/cm^2^) >300 Ω/cm^2^. TER was measured across Caco-2 monolayers as an estimate of tight junctional integrity using an epithelial volt-ohmmeter (EVOM) and chopstick attachment (Millipore, Burlington, MA, USA). On the day of the assay, monolayers were either cultured with UCC118™ at 1 × 10^8^ CFU/mL alone or left untreated. TER was measured prior to commencement of treatments, following pretreatment, and then at 2 h intervals thereafter. Change in TER is represented as a percentage of original TER.

### 2.9. Immunoblot

Western Immunoblot analysis was performed on colon lysates extracted by mechanical homogenisation in RIPA buffer supplemented with protease inhibitors (Roche, Basel, Switzerland). Samples were clarified, denatured in sample buffer and boiled for 5 min. Protein lysate was fractionated on 10% SDS-PAGE, transferred to polyvinylidene fluoride membranes (Millipore) and probed with primary antibody to IL-10 (1:500 dilution, Proteintech, Rosemont, IL, USA) or murine β-actin (1:1000 dilution, Clone AC-74, Sigma), incubated with horseradish peroxidase-conjugated secondary antibodies (Santa Cruz Biotechnology, Dallas, TX, USA) and visualised using ECL HRP substrate (ThermoFisher, Waltham, MA, USA), GelAS 4000 and ImageLab software.

### 2.10. LPS-Stimulation of Bone Marrow-Derived Macrophages

BMDMs were isolated from the tibia and femur of age and sex-matched mice. Briefly, bone marrow was extracted and resuspended in RBC-lysis buffer for 3 min, followed by the addition of complete DMEM and centrifugation. The white cell pellet was resuspended in complete DMEM and passed through a 100 μm cell strainer to filter out debris. The cell suspension was cultured for 6 days in complete DMEM supplemented with 20% L929 conditioned media for differentiation. BMDMs were removed by scraping and seeded into 12 well plates at 5 × 10^5^ cells/well in antibiotic-free DMEM with 20% L929 conditioned media. BMDMs were pre-treated with PBS or 1 × 10^8^ CFU/mL of UCC118™. LPS was used to stimulate BMDMs at 100 ng/mL for 2 h. Cells were harvested for gene expression analysis, and supernatant was used for ELISA analysis.

### 2.11. FITC–Dextran Assay for Barrier Permeability

FITC–Dextran (4 kDa and 40 kDa) solution was made at a concentration of 80 mg/mL in sterile PBS in light-safe tubes. Mice were gavaged with 150 μL of FITC–Dextran solution and food was removed from the cages. After 4 h, mice were euthanised and blood was harvested in pre-weighed tubes and kept on ice. Then, 15% *v/v* acid–citrate–dextrose solution was added to each tube as an anti-coagulant and centrifuged at 5000 rpm for 10 min. Plasma was transferred to a new tube, diluted 1:10 and used for measurement of fluorescence (excitation at 485 nm and absorbance at 530 nm). FITC–Dextran 4 kDa was used to assess barrier permeability at baseline in healthy mice, and FITC–Dextran 40 kDa was used in colitic mice.

### 2.12. Enzyme Linked Immunosorbent Assay (ELISA)

Lysates from mouse colonic tissue and supernatants from murine bone marrow-derived macrophages were assayed for the presence of IL-10 (Invitrogen # 88-7105-88), IL-1β (Invitrogen #88-7013-77) and IL-6 (Invitrogen #88-7064-77) as per the manufacturer’s protocol. The optical density at 450 nm of samples was measured, and cytokine concentrations were determined using a standard curve as pg/mL. For colonic tissue, tissue weights were used to normalise lysate concentrations.

### 2.13. 16s rRNA Sequencing for Microbiota Analysis

DNA extraction was performed from faecal samples using a commercial kit according to the manufacturers’ instructions. The 16S rRNA gene (V3–V4 region) was PCR amplified following the Illumina 16S Sample Preparation Guide using the Universal primers. The resulting amplicons were sequenced on the Illumina MiSeq platform using v3 sequencing chemistry with 2 × 250 pb paired-end reads. Sequences were filtered on the basis of quality (removal of low quality nucleotides at the 3′ end, and remove windows 20 nt with a low average quality) and length (removal of sequences with less than 200 nt) with prinseq-lite, and the paired-end reads with a minimum overlap of 20 bp were joined using Fastq-join. The sequences were also cleaned of dereplicates and unique sequences and chimeras were eliminated against the gold database using the closed-reference Usearch v7.0 algorithm. The resulting sequences were clustered with 97% identity level (calculated at the operational taxonomic unit; OTUs) using the closed-reference Usearch v7.0 algorithm. The taxonomic assignment of these OTUs was obtained against the Ribosomal database project classifier. Alpha and beta-diversity was determined using QIIME, and additional analyses were performed with the R package phyloseq.

### 2.14. Statistical Analysis

Numerical results are given as arithmetic means ± standard error of the means. Statistical differences were analysed by GraphPad Prism 6.0 statistical software (GraphPad Software Inc., San Diego, CA, USA). *p*-values of 0.05 or less (*p* ≤ 0.05) are considered statistically significant. Then, 16s rRNA sequencing data were analysed by Adonis for beta-diversity analysis. ANOVA was used to calculate the significance in the analysis of the alpha-diversity index. Statistical differences between multiple samples were estimated by Kruskal–Wallis and adjustment of the *p*-value by the Benjamini–Hochberg method, while the comparisons between groups individually was performed using the Mann–Whitney U test. Statistical significance was established at *p* ≤ 0.05 (*), *p* ≤ 0.01 (**) and *p* ≤ 0.001 (***).

## 3. Results

### 3.1. Lactobacillus Salivarius UCC118™ Reduces the Severity of DSS-Induced Colitis in Mice

**Figure 1:**
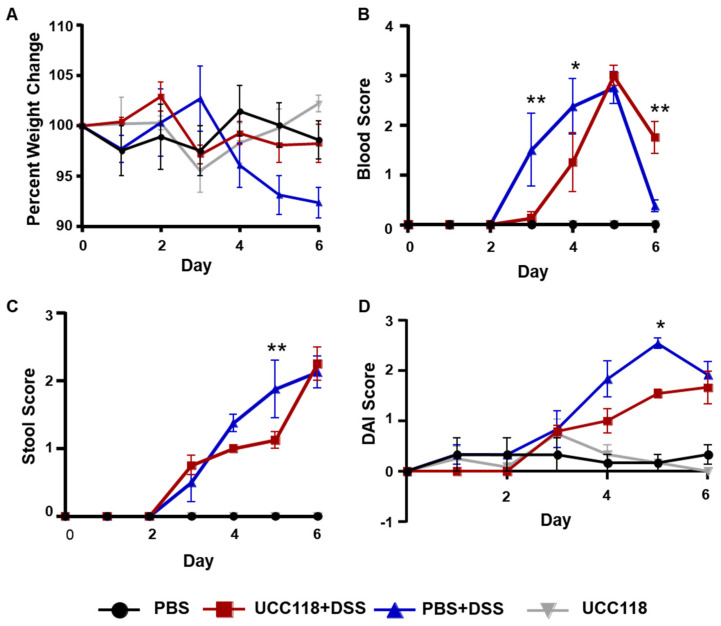
Pre-treatment with UCC118^TM^ reduces severity of disease in a DSS-induced colitis model. C57BL/6JOlaHsd mice were pre-treated with 100 μL UCC118™ (10^9^ CFU/mouse) or sterile PBS by oral gavage daily. On day 7, mice were treated with 2.5% dextran sulphate–sodium via drinking water *ad libitum* for 6 days. Mice were weighed daily (**A**) and stool was checked for occult blood (**B**) and diarrhoea (**C**). Disease activity index (DAI, **D**) was plotted as an average of weight score, blood score and stool score on a scale of 0–4. Representative results of two independent experiments. Statistical analysis performed by two-way ANOVA. Stars indicate statistical significance in comparisons between PBS + DSS and UCC118 + DSS groups. ** *p* < 0.01, * *p* < 0.05. *n* = 4 mice per group.

UCC118™ has been shown to have a significant probiotic benefit against bacterial infection; however, its use in mouse models of intestinal inflammation remains poorly studied [12,15]. We first assessed the potential of daily UCC118™ administration to protect against DSS-induced colitis in mice. For one week prior to DSS treatment, mice were gavaged daily with either sterile PBS or UCC118™. Previous studies have shown that UCC118™ survives the acidic environment of the stomach, and viable bacteria can be detected in the faecal contents [9]. Mice pretreated with UCC118™ were effectively protected from DSS-induced weight loss (Figure 1A) and showed a trend towards reduced rectal bleeding (Figure 1B) and diarrhoea (Figure 1C) in the early stages of DSS treatment. Overall, the disease activity index of UCC118™ pre-treated mice was significantly lower than the PBS mice on day 5 of DSS treatment (Figure 1D), though no difference was observed by end of the treatment. A similar trend of protection in early stages of DSS-induced colitis was observed in an independent experiment. At the tissue level, there was no significant difference in colon length or colonic myeloperoxidase activity between the two treatment groups (Supplementary Figure S1). Histological analysis was performed to assess signs of immunopathology in colon sections of mice. As expected, control mice displayed no inflammation. In the DSS treatment groups, PBS mice had a slight trend towards increased tissue damage compared to UCC118™ mice; however, cell infiltration scores were similar across both groups (Supplementary Figure S1). Taken together, these findings demonstrate that treatment with UCC118™ offers partial protection against the development of inflammation in a DSS-induced colitis model.

### 3.2. Lactobacillus salivarius UCC118™ Does Not Affect Barrier Integrity In Vivo

**Figure 2:**
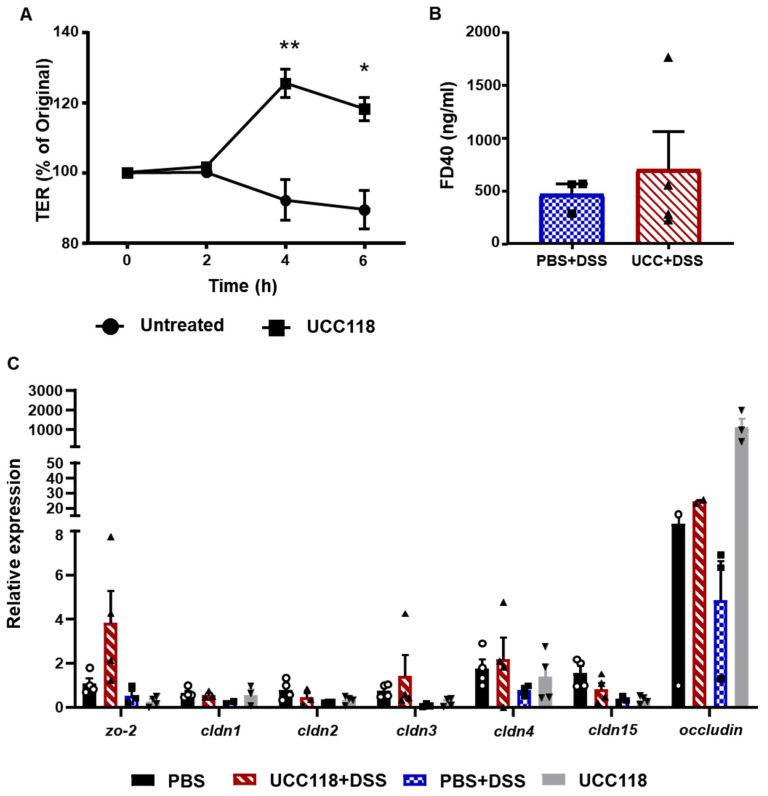
Pre-treatment with UCC118^TM^ does not affect barrier integrity in a DSS-induced colitis model. (**A**) Differentiated Caco-2 monolayers on transwell inserts were treated with PBS or UCC118™ (10^8^ CFU/well) and transepithelial resistance (TER) was measured. Data are plotted as the average of three wells per group. Representative graph of two independent experiments. Statistical analysis was performed using *t*-test. * *p* < 0.05, ** *p* < 0.01. (**B**) Barrier permeability in the DSS-colitis induced mice pre-treated with PBS or UCC118™ was measured using FITC–Dextran (40 kDa) levels in the serum. (**C**) Mouse colon tissue of control and DSS-induced colitis were analysed for expression of key tight junction proteins; *n* = 4 mice per group; symbols represent individual mice.

To determine the mechanism underlying the observed UCC118™-mediated protection, we assessed the effect of UCC118™ on intestinal epithelial cells. As a first step, we used differentiated Caco-2 cell monolayers to study whether treatment with *Lactobacillus salivarius* UCC118™ had any effect on transepithelial electrical resistance (TER). Permeability (TER) across differentiated Caco-2 monolayers was measured using an epithelial voltohmeter at 2 h intervals. It was found that treatment with UCC118™ strain alone resulted in a steady increase in TER over time, which peaked at 4 h (Figure 2A). This observation is in line with the study by Miyauchi et al., which observed that UCC118™ suppressed H_2_O_2_-mediated loss of TER in Caco-2 cells [13]. To test whether UCC118™ could boost barrier integrity in vivo, we performed the treatment of mice with UCC118™ or PBS for 7 days and assessed barrier permeability using FITC–Dextran (4 KDa). Treatment with UCC118™ did not alter body weight, colon length or barrier permeability to FITC–Dextran (Supplementary Figure S2). Expressions of tight junction proteins in colon tissue were also unaffected (Supplementary Figure S2). Barrier function was also assessed after DSS-treatment in the PBS + DSS and UCC118 + DSS groups using FITC–Dextran (40 KDa). No significant difference in barrier permeability was observed between the two groups (Figure 2B). The expression of tight junction proteins *zo-2*, *cldn-3, occludin* and *cldn-4* appeared to be increased in UCC118 + DSS mice compared to PBS + DSS and PBS controls; however, this was not statistically significant (Figure 2C). This suggests that administration of UCC118™ may reduce the extent of the inflammation-induced disruption of epithelial tight junctions.

### 3.3. Lactobacillus salivarius UCC118™ Promotes IL-10 in a DSS-Colitis Model

**Figure 3:**
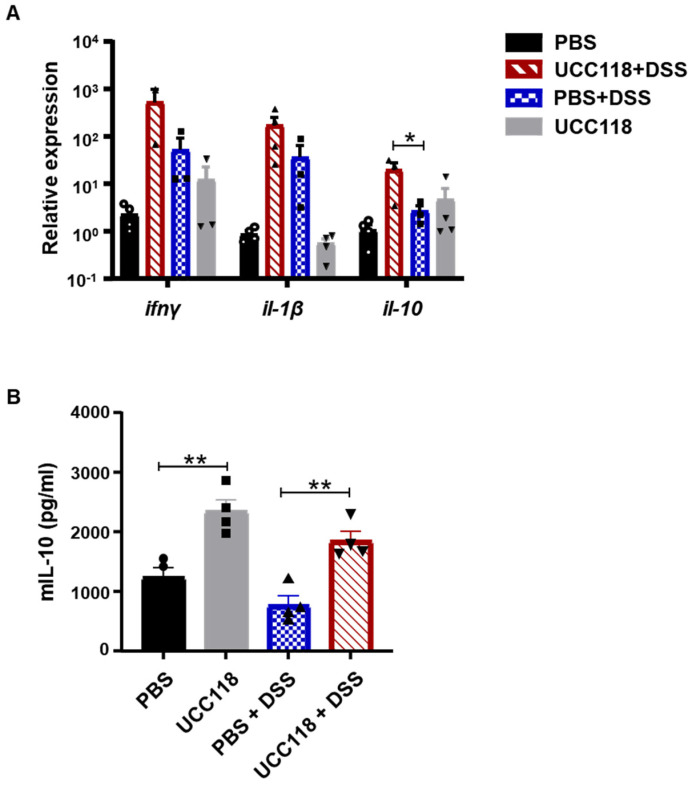
Pre-treatment with UCC118^TM^ promotes IL-10 production in a DSS-induced colitis model. Mouse colon tissue on day 6 of DSS-induced colitis were analysed for expression of key cytokines (**A**). IL-10 protein levels in the colon were measured by ELISA (**B**). *n* = 4 mice per group, symbols represent individual mice. Statistical analysis was performed using one-way ANOVA. * *p* < 0.05, ** *p* < 0.01.

We hypothesised that the protective effect of UCC118™ against intestinal inflammation might be mediated by the mucosal immune response. Colonic tissue of the mice from control and DSS treatment groups was analysed for the expression of key cytokines. Interferon-γ and interleukin-1β expression were similarly expressed in the PBS +DSS and UCC118 + DSS groups; however, interleukin-10 expression was significantly upregulated by UCC118™ compared to PBS (Figure 3A). A similar increase in IL-10 gene expression was also observed at baseline after 7 days of UCC118™ treatment, though IL-10 levels measured by ELISA remained unchanged (Supplementary Figure S2). IL-10 protein levels in the DSS-treated cohort were determined by ELISA. ELISA showed a significant upregulation of IL-10 in the UCC118™-alone mice compared to PBS control mice (Figure 3B). Similarly, UCC118 + DSS mice showed higher levels of IL-10 in colonic tissue compared to PBS + DSS mice (Figure 3). These results suggest that the protective effect of UCC118™ against colitis might derive from the upregulation of the anti-inflammatory cytokine IL-10.

### 3.4. Lactobacillus salivarius UCC118™ Promotes M2 Phenotype in Bone Marrow-Derived Macrophages

**Figure 4:**
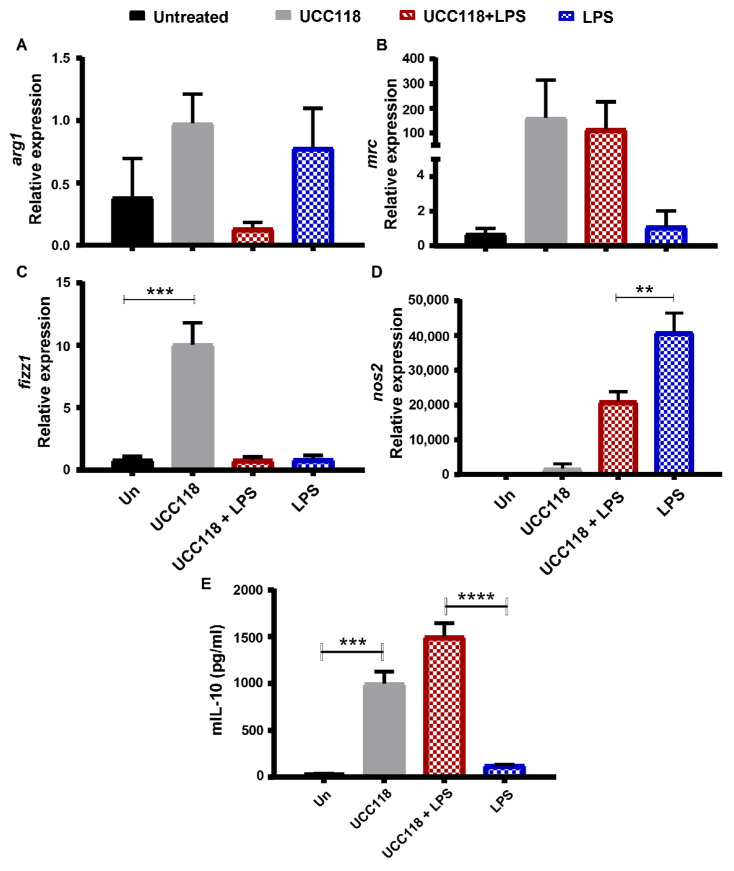
UCC118^TM^ promotes M2-like phenotype and IL-10 production in bone marrow-derived macrophages. Bone marrow-derived macrophages were cultured from C57BL/6JOlaHsd mice and cultured for 6 days in complete DMEM containing L929 conditioned media. BMDMs were pre-treated with vehicle control or UCC118™ (10^8^ CFU/well) for 2 h followed by stimulation with LPS (100 ng/mL) for 4 h. Gene expressions of M1 and M2 macrophage markers (**A**) *arg1* mRNA expression, (**B**) *mrc* mRNA expression, (**C**) *fizz1* mRNA expression, (**D**) *nos2* mRNA expression, and (**E**) mIL-10 protein were analysed by qRT-PCR and ELISA respectively. Representative data of *n* = 3 biological replicates. Statistical analysis by one-way ANOVA. ** *p* < 0.01, *** *p* < 0.001, **** *p<* 0.0001.

The increased level of IL-10 in the UCC118™-treated colitic mice suggests that the probiotic might have an anti-inflammatory effect in vivo. These observations are in line with a study in a mouse model of rheumatoid arthritis where treatment with UCC118™ reduced inflammation and upregulated IL-10 production [16]. In a study of MLN mononuclear cells from colitis patients by O’Mahony et al., incubation with UCC118™ resulted in a similar increase in IL-10 secretion [17]. We postulated that this anti-inflammatory action of UCC118™ might be the result of the effect of the probiotic on immune cells in the colonic lamina propria, specifically macrophages. To test this hypothesis in vitro, we used murine bone marrow-derived macrophages (BMDMs) as a model. Naïve or LPS-treated BMDMs were incubated with UCC118™. Treatment of naïve BMDMs with UCC118™ significantly upregulated the expression of M2 macrophage markers *arg1*, *mrc* and *fizz1* (Figure 4A–C) and was associated with an increase in IL-10 secretion (Figure 4E). In LPS-stimulated BMDMs, UCC118™ treatment upregulated the expression of M2 marker gene *mrc*, downregulated the M1 macrophage marker gene *nos2* (Figure 4A–D) and was again associated with significantly higher levels of IL-10 secretion compared to the LPS-only control (Figure 4E). These results suggest that UCC118™ promotes an M2-like phenotype in BMDMs to increase the production of IL-10.

In the colon tissues from mice treated with UCC118™ for 1 week, levels of M2 and M1 marker gene expression were similar to that of the control mice (Supplementary Figure S3). However, in the DSS-colitis mice, a significantly higher expression of M2 marker *fizz1* was observed in UCC118 + DSS mice compared to PBS + DSS mice (Supplementary Figure S3). A trend for higher expression of *mrc* and *arg1* was also observed in the UCC118 + DSS group. Surprisingly, *nos2* expression was also significantly higher in the UCC118™-treated group compared to DSS-only controls (Supplementary Figure S3). These results suggest that UCC118™ pre-treatment might regulate M2-like marker expression to increase IL-10 secretion and dampen inflammation in the DSS-colitis model.

### 3.5. Lactobacillus salivarius UCC118™ Promotes Recovery from DSS-Induced Colitis

**Figure 5:**
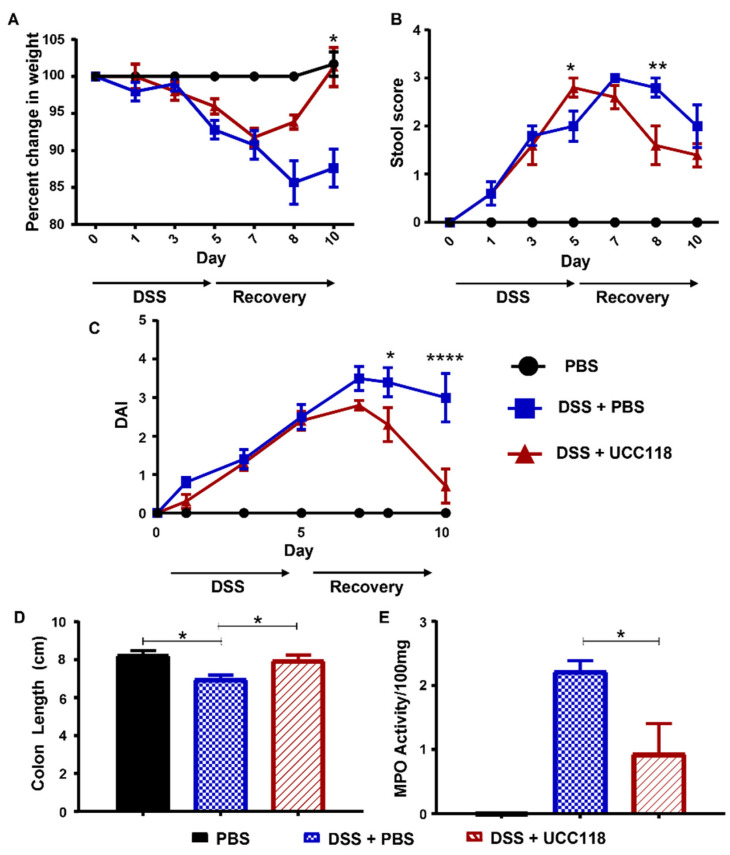
UCC118^TM^ provides therapeutic benefit in a recovery model of DSS-induced colitis model. C57Bl/6JOlaHsd mice were administered 2.5% DSS *w/v* ad libitum for 5 days to induce colitis. On day 6, the DSS was removed and replaced with water for a further 5 days to allow recovery. During recovery, mice were given either UCC118™ or sterile PBS by oral gavage. Mice were scored for development of disease: weight (**A**), diarrhoea (**B**), disease activity index (**C**). After sacrifice, colon length (**D**) was measured and myeloperoxidase (MPO) activity (**E**) determined. n ≥ 5 mice per group, *n* = 3 mice in the water control, mean values ± SEM are presented. Statistical analysis by two-way ANOVA. * *p* < 0.05, ** *p* < 0.01, **** *p* < 0.0001.

**Figure 6:**
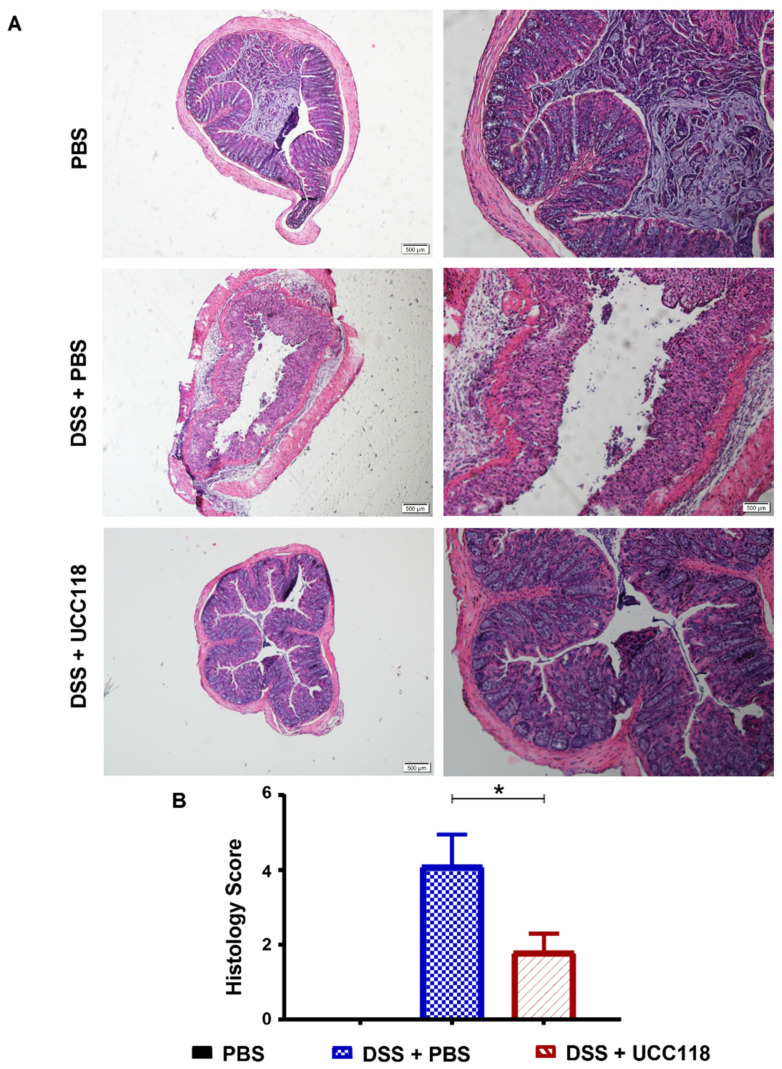
UCC118^TM^ promotes recovery from tissue damage and immune infiltration in a DSS-induced colitis model. Colon sections of mice treated for 5 days with 2.5% DSS ad libitum, followed by 5 days with regular drinking water with or without UCC118™ and control group (water only) were analysed using haematoxylin and eosin (H&E) staining. (**A**) Colitis severity was assessed by combined histological score of tissue disruption and colon cellular infiltration (**B**). Histology score represented as mean values ± SEM of *n* = ≥3 mice per group. *p* values were calculated using Student’s *t*-test, * *p* < 0.05.

Given the anti-inflammatory effect of UCC118™ in vitro as well as in the pre-treatment model of colitis, we next sought to determine whether the UCC118™ strain could offer any therapeutic effect in already established colitis. A DSS-recovery model was employed to determine whether administration of UCC118™ during recovery from DSS-induced colitis could enhance recovery and restore the intestinal epithelium. As expected, all groups of mice experienced disease symptoms at a relatively uniform rate during induction of colitis with DSS (Figure 5A). Following the removal of DSS and return to normal drinking water, mice received UCC118™ or PBS by oral gavage. During this recovery period, mice administered UCC118™ exhibited accelerated recovery as evidenced by increased appetite, increased weight gain (Figure 5A), reduced diarrhoea (Figure 5B) and a reduction in the overall disease activity (Figure 5C) compared to PBS-treated mice. Faecal blood scores were not included in the calculation of DAI, as occult blood proved undetectable across both treatment groups once DSS was removed. Following termination of the trial, colon lengths were measured and found to be significantly increased in mice which received UCC118™ during the recovery period compared to those recovering from DSS with PBS administration (Figure 5D). Interestingly, there was no significant difference in colon length between mice receiving UCC118™ strain and the water control mice. MPO activity appeared to be lower in UCC118™-treated mice compared to PBS-treated mice, which was further indicative of reduced inflammation (Figure 5E). Histological analysis showed that, as expected, water control mice displayed no inflammation. DSS + PBS mice had significantly more inflammation in colonic tissue compared to DSS + UCC118 mice, as indicated by a cumulative histology score generated from crypt damage and inflammatory infiltrate scores (Figure 6A,B).

### 3.6. Lactobacillus salivarius UCC118™ following Induction of DSS-Colitis Does Not Induce IL-10

**Figure 7:**
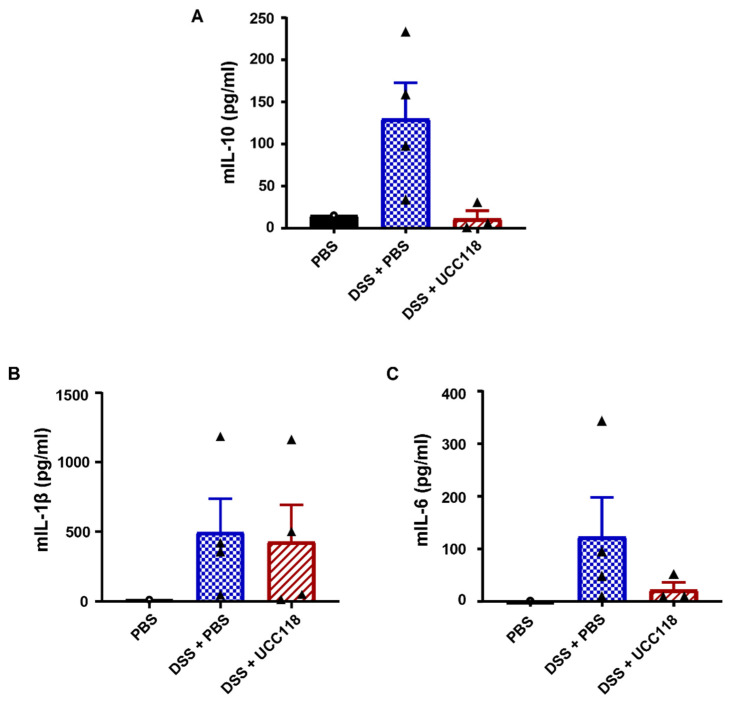
Cytokine response is not responsible for accelerated recovery after UCC118^TM^ treatment in a DSS-induced colitis model. IL-10 levels in colonic tissue of mice treated with DSS followed by PBS or UCC118™ treatment during recovery phase were analysed by ELISA (**A**–**C**): IL-10 (**A**), IL-1β (**B**) and IL-6 (**C**). Four mice per group for ELISA analysis. Statistical analysis by one-way ANOVA.

UCC118™ was seen to be highly effective as a therapeutic to promote recovery from DSS-induced colitis. We hypothesised that UCC118™-induced IL-10 might be responsible for this effect. IL-10 levels as measured by ELISA showed an opposite trend compared to expectations, with lower levels of IL-10 in colonic tissues of DSS mice treated with UCC118™ compared to DSS + PBS mice (Figure 7A). ELISA analysis showed that while levels of inflammatory cytokine IL-1β were significantly higher in colitis mice compared to healthy controls, no significant difference was seen between the UCC118™ and PBS-treated colitis mice (Figure 7B). UCC118™ treatment resulted in lower IL-6 levels in the colon compared to DSS + PBS mice, though the trend did not reach statistical significance (Figure 7C). These results suggest that the therapeutic benefit of UCC118™ in the DSS-colitis recovery model is independent of IL-10.

### 3.7. Administration of Lactobacillus salivarius UCC118™ following Induction of DSS-Colitis Produces a Distinct Gut Microbiota

**Figure 8:**
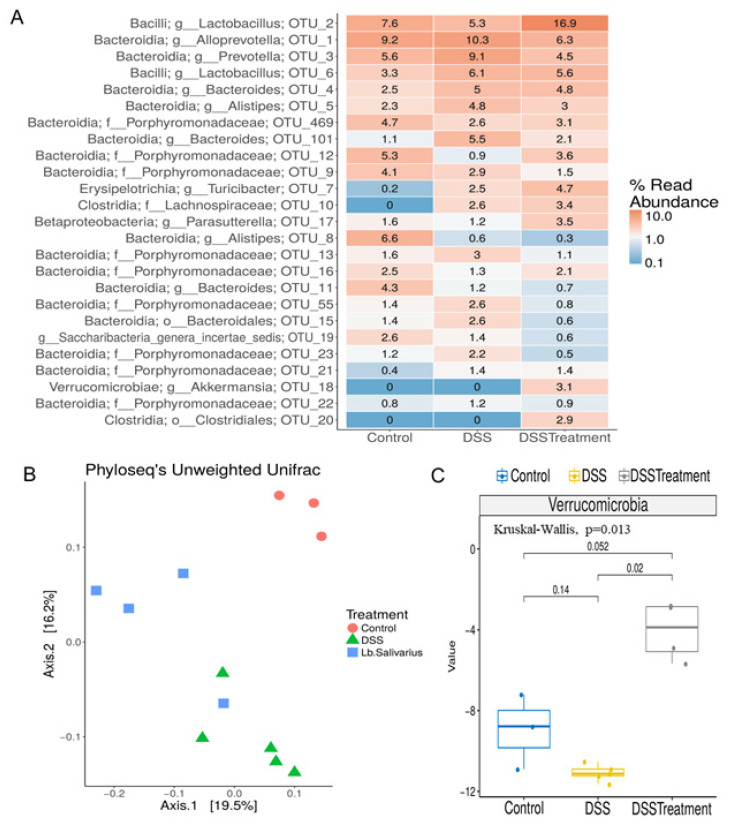
UCC118^TM^ treatment promotes recovery of the faecal microbiome in a DSS-induced colitis model. (**A**–**C**) The microbiota composition of faecal samples harvested from mice recovering from DSS-colitis with or without UCC118™ treatment (DSS-Treatment or DSS-PBS, respectively) were analysed by MiSeq 16s rRNA sequencing and compared with water control mice. (**A**) Sequence reads were compared to the RDP database and compared for OTU analysis. (**B**) Comparison of diversity between mouse groups: Control (red), DSS-PBS (green), and UCC118™-treated (blue). (**C**) Kruskal–Wallis analysis of significant differences at phylum level. Results shown are representative of biological groups n ≥ 3, mean values ± SD are presented. Statistical analysis using unpaired T-test.

Our data suggest that UCC118™ promotes recovery from colitis in an IL-10 independent mechanism. We wanted to investigate if other mechanisms might be involved in this therapeutic effect. UCC118™ has previously been used as a probiotic in both rodent and animal models [10,18]. UCC118™ has been shown to reach the intestine and retain viability in the gastrointestinal tract. Studies of probiotic administration of UCC118™ in healthy mice have shown that the probiotic has a relatively subtle effect on the mouse microbiome, affecting a few genera in the Bacteroidetes and Firmicutes phyla. The bacteriocin produced by UCC118™ has been implicated in this effect [18]. Therefore, we assessed whether the administration of *Lactobacillus salivarius* UCC118™ to mice following induction of colitis could restore any changes to the microbiota as a result of inflammation. 16S rRNA sequencing of faecal samples was employed to investigate the differences in the composition of the microbiota between *Lactobacillus salivarius* UCC118™-treated or PBS control mice following recovery from DSS-colitis. After quality filtering and length trimming, an average of 134,490.7 (±24,288.52 SD) 16S rRNA high-quality sequences were generated per sample. These corresponded to three different groups: Control (N = 3), DSS-PBS (N = 5), DSS-Treatment (N = 4). The most abundant phyla found in these samples were *Firmicutes, Bacteroides*, *Candidatus Saccharibacteria*, *Proteobacteria* and *Actinobacteria*. The more abundant families detected were *Porphyromoandaceae, Lactobacillaceae, Prevotelalceae*, *Bacteroidaceae*, *Lachnospiraceae*, *Rikenellaceae*, *Ruminococcaceae* and *Erysipelotrichaceae*. At genus level, the most abundant were *Lactobacillus,* as well as *Alloprevotella, Bacteroides*, *Alistipes*, *Prevotella*, *Parasutterella*, *Turicibacter*, *Saccharibacteria*, *Barnesiella* and *Akkermansia*. Each group showed quite varied assignments, indicating a distinct microbial profile for each. This distinction in bacterial composition was evident at the 25 most abundant operational taxonomic units (OTUs) shown in Figure 8A, indicating each OTU with their respective genus and phylum name (Figure 8A). The alpha diversity did not differ significantly across groups (*p* > 0.05, 2-way ANOVA). However, significant (*p* value = 0.001) differences in beta-diversity were evident between all groups, with a clear phylogenetic separation between all groups as represented by Principal Coordinates Analysis (PcoA) following the clustering of all reads at 97% similarity (Figure 8B). *Akkermansia* emerged as being the phylum with the most striking differences between groups, being significantly more abundant in the DSS-Treatment group (Figure 8C). As was expected due to the association of DSS-models with causing dysbiosis, the most striking differences are evident between control samples compared with the two DSS-recovery groups. In the control group, we see higher *Alistipes*, *Bacteroides*, *Porphyromonadaceae* and *Saccharibacteria genera incertae sedis* and a lower proportion of *Clostridia*, *Lachnospiraceae*, *Erysipelotrichia*, *Turicibacter* and *Bacteroides* compared with groups recovering from DSS. Overall, this indicates that DSS treatment and recovery is accompanied by a shift in microbial composition. The control group also contained a higher proportion of *Bifidobacteriaceae* and *Roseburia* compared to the other two groups, which are two genera known to generally indicate good GI health (Supplementary Figure S4). Despite the chaotic shift in microbiota noted following DSS treatment, upon examining these distinctions further, it became obvious that *Lactobacillus salivarius* UCC118™-recovery mice produced a highly distinct bacterial profile when compared with DSS-mice recovering with PBS (DSS-PBS). In mice recovering from DSS with *Lactobacillus salivarius* UCC118™ treatment (DSS-Treatment), we observed a higher proportion of *Lactobacillus, Verrucomicrobia*, *Akkermansia and Clostrdia XIVa* genera and a lower proportion of *Alloprevotella, Prevotella, Alistipes, Bacteroides* and *Porphyromonadaceae* when compared with the DSS-PBS group. In the DSS-PBS group, we observed a higher proportion of *Alloprevotella*, *Turibacter*, *Prevotella*, *Alistipes*, *Bacteroides* and *Porphyromonadaceae* and a lower proportion of *Lactobacillus*, *Akkermansia* and *Porphyromonadaceae* compared to control and DSS-Treatment groups (Supplementary Figure S4). Lastly, if we focus on comparing the control group against DSS-Treatment, we do not find significant differences, indicating that those mice recovering with *Lactobacillus salivarius* UCC118™ may experience a more efficient return to microbiota homeostasis.

## 4. Discussion

To be classed as a probiotic, a bacterial strain should fulfil the definition outlined by the World Health Organisation as follows: live micro-organisms which, when administered in adequate amounts, confer a health benefit on the host [19]. Until recently, it remained to be shown definitively if this characteristic was indeed shared by the UCC118™ strain. An important study by Corr et al. demonstrated that UCC118™ protects mice against infection with *Listeria monocytogenes* and furthermore identified the bacteriocin Apb118 as the underlying mechanism responsible for this protection [11]. Although a previous study indicated potential for UCC118™ strain in an IL-10-deficient mouse model of chronic colitis [15,20], it has remained unclear until now whether *Lactobacillus salivarius* UCC118™ could be a good candidate for the treatment of IBD. In the present study, we demonstrate the disease-altering effects of UCC118™ in a model of DSS-induced colitis. Our study evaluates both the prophylactic as well as the therapeutic properties of this strain in the context of acute intestinal inflammation. We observe that the benefits conferred by UCC118™ differ in each of these regimens. When used prophylactically, UCC118™ gives modest protection against the development of DSS-induced colitis but significantly accelerates recovery from colitis when administered therapeutically. Our results, specifically in the recovery model, are in contrast with the study by Feighery et al., where UCC118™ failed to have any beneficial effects in both an IL-10 knockout mice model as well as in a DSS-colitis model [12]. Differences in the observed disease outcome might be attributed to different feeding regimens and routes adopted by their group.

In vitro studies by our group as well as others have shown that UCC118™ has a barrier protective effect. Treatment with UCC118™ results in an increase in transepithelial resistance across cell monolayers, both in healthy monolayers as well as in the context of inflammatory insult [13]. Lomasney et al. also observed an increase in colonic TER using the Ussing chambers method in mice fed UCC118™ for 2 weeks, though no change in tight junction proteins was observed [21]. In contrast, no such barrier protective effect was observed in vivo in this study. UCC118™ pre-treatment did not alter barrier permeability as measured by the FITC–Dextran method or the expression of key tight junction proteins in the colon at baseline or during acute colitis. This suggests that the observed protection by UCC118™ is not mediated via changes in the gut barrier.

UCC118™ pre-treatment was associated with a significant upregulation of IL-10 levels both at mRNA and protein level at baseline as well as during colitis. We hypothesised that this effect might be mediated by the reprogramming of colonic immune cells by UCC118™. To test this in vitro, we treated bone marrow-derived macrophages with UCC118™ in the presence and absence of LPS as an inflammatory stimulus. UCC118™ exposure was observed to produce an M2-type phenotype in macrophages, with upregulation of M2 differentiation markers such as Fizz1, downregulation of M1 marker Nos2 and increase in IL-10 secretion. These observations are corroborated by a recent study by Udayan et al., where UCC118™ stimulated IL-10, TNFα, IL-12 and IL-6 production by macrophages in a TLR2-independent but MyD88-dependent manner [22]. Similar observations have also been made for UCC118™ treatment in murine bone marrow-derived dendritic cells [23].

The upregulation of M2 markers in BMDMs treated with UCC118™ was accompanied by a downregulation of Nos2 by UCC118™ in naïve BMDMs as well BMDMs treated with LPS. Surprisingly, this trend was not observed in vivo in the DSS-pretreatment model. UCC118 + DSS mice had a significantly higher gene expression of *nos2* compared to PBS + DSS mice. Nos2 is considered to have a classic pro-inflammatory action that aggravates inflammation in diseases such as IBD [24]. Recent studies have highlighted that the role of Nos2 in the context of colitis is complex, with Nos2 activity being beneficial for epithelial homeostasis [25,26] as well as during DSS colitis [24,27]. Varying results of Nos2 inhibition have been obtained depending on the nature of the intervention (gene deletion models vs. chemical treatment) [28,29] as well as its timing [30]. Further study is required to unravel the link between UCC118™ and Nos2 and determine if Nos2 is involved in the beneficial effects of UCC118™ in colitis.

The anti-inflammatory activity of UCC118™ has also been observed in immune cells isolated from patients with active colitis [17]. UCC118™ was found to specifically affect immune cells from the mesenteric lymph node and not peripheral blood. MLN cells, both from inflamed and non-inflamed sites in the patient bowel, produced IL-10 in response to UCC118™ treatment. Interestingly, this increase in IL-10 production after UCC118™ exposure was also observed in dendritic cells isolated from MLN and peripheral blood. These results complement our findings of macrophage reprogramming by UCC118™. Supplementation of UCC118™ to healthy mice has also shown to skew the intestinal immune response, in both Peyer’s patches and the small intestine, towards an immune regulatory phenotype [31]. This effect was a combination of increased frequency of regulatory T cells as well as dendritic cells in the gut. This suggests that the source of the observed IL-10 upregulation in the UCC118™ pre-treatment model might be a combination of macrophages, dendritic cells and T cells in the colon. Surprisingly, we observed no significant changes in IL-10 levels upon UCC118™ supplementation in the recovery model. In fact, IL-10 levels were significantly lower in UCC118™-fed mice compared to PBS control mice recovering from colitis. Assessing IL-10 levels at earlier stages of the recovery might be helpful in clarifying whether the kinetics of IL-10 induction are different in these two groups or if the protection observed is IL-10 independent.

Having noted a significant therapeutic role for UCC118™ in established colitis, we were interested in examining the profile of the microbiota in mice following recovery from disease in order to identify changes suggestive of remission and return to homeostasis. While a previous study demonstrated that UCC118™ strain did not significantly alter mouse microbiota composition basally [18], we chose to examine alterations in the face of disease recovery—a situation where microbiota repletion would be most beneficial to the host. DSS-induced colitis results in a chaotic shift in microbiota composition. While DSS indeed perturbed microbiota composition in the current study, recovery with UCC118™ produced a very distinct microbial profile compared with PBS-recovery mice. This demonstrates that the strain influences the microbiota in a manner that may promote healing and protect against the further perpetuation of disease. As expected, a greater abundance in *Lactobacillus* spp. was observed in UCC118™-treated group. Importantly, in the UCC118™-treated mice, we found high proportions of *Coprococcus* (*p*-value-corrected = 0.001), a major producer of short-chain fatty acids (SCFA), namely butyrate [32]. Many other SCFA-producers were identified in the UCC118™-treated recovery group; for example, one particular phlyum that emerged was the *Verrucomicrobiaciae.* This phylum has been shown to differ in the faecal microbiota of IBD patients in remission versus in active disease, generally being associated with remission [33]. This is interesting in that it reflects a return to homeostasis for the microbiota in the presence of UCC118™. *Akkermansia*, a genus identified to be higher in UCC118™-treated recovery mice compared with PBS recovery mice, is typically seen to be beneficial to the host and can promote epithelial tight junction integrity [34]. *Clostridiaciae*, encompassing a variety of bacterial clades with divergent effects on health and disease, were lower at an overall genus level; however, the cluster Closdridia XIVa were seen to be higher in UCC118™-treated recovery mice: This cluster is an important producer of the SCFA butyrate, which promotes gastrointestinal health [34]. Within the DSS-alone (PBS-treated) group, other butyrate producers such as *Roseburia* were seen to be diminished, while UCC118™-treated mice seem to recover this population, with no significant difference seen compared with the control mice. Furthermore, UCC118™-treated recovery mice exhibited a higher ratio of *Firmicutes* compared with *Bacteroides* compared with DSS-alone (PBS-treated) recovery mice. An abundance of *Firmicutes* is generally seen as favourable in terms of disease prevention.

## 5. Conclusions

In summary, this study reveals a role for *L. salivarius* UCC118™ in the prevention and treatment of intestinal inflammation. The beneficial effects of UCC118™ were observed during use as a prophylactic as well as a therapeutic, with high effectiveness as the latter. Mechanistically, UCC118™ acted through the upregulation of IL-10, most likely by promoting an anti-inflammatory bias in local macrophages. UCC118™-mediated IL-10 induction was effective at delaying the onset of colitis; however, its action during the recovery phase of colitis was IL10-independent. Furthermore, we reveal the ability of UCC118™ strain to promote the resolution of the immunopathology caused by activation of disease as well as in recovery of the microbiota. UCC118™ not only promoted the return of the colitis microbiome to a healthy state but was also seen to increase the abundance of beneficial taxa beyond levels originally observed during health. Together, these findings suggest a promising potential application for this *L. salivarius* UCC118™ as a therapeutic in IBD. 

## Figures and Tables

**Table 1:** Primers used for qRT-PCR.

Gene	Primer Sequence
ZO-2	FP: GCACCCTGACATCTATGCG
	RP: CACTGCCGTAGCTTCCTCTG
Claudin 1	FP: ATGACCCCTTGACCCCCATC
	RP: GGAGCAGGAAAGTAGGACACC
Claudin 2	FP: CCTGGGATTGTGCTTGAGGT
	RP: TGACCCCCATCTACCACAGA
Claudin 3	FP: CCGCAAGGACTACGTCTGAG
	RP: CAAGCAGACTGTGTGTCGTCT
Claudin 4	FP: GTAGAGTGGATGGACGGGTT
	RP: CATTAGCAAGACAGTGCGGA
Claudin 15	FP: GGACCCTCCACATACTTGCT
	RP: GCACTCCAGCCCAAGTAGAG
ZO-1	FP: CAAAGCCCACCAAGGTCAC
	RP: TCTCTTTCCGAGGCATTAGCA
Occludin	FP: CAGGGCTCTTTGGAGGAA
	RP: TACACGATCGTGGCAATAAAC
IL-10	FP: TTGAATTCCCTGGGTGAGAAG
	RP: TCCACTGCCTTGCTCTTATTT
IFNgamma	FP: CAGGCTGTCCCTGAAAGAAA
	RP: CATTCGGGTGTAGTCACAGTT
IL-1beta	FP: TTCAGGCAGGCAGTATCACTC
	RP: GAAGGTCCACGGGAAAGACAC
Arg1	FP: TTTTAGGGTTACGGCCGGTG
	RP: CCTCGAGGCTGTCCTTTTGA
Fizz1	FP: ACTGCCTGTGCTTACTCGTTGACT
	RP: AAAGCTGGGTTCTCCACCTCTTCA
MRC	FP: GGCGAGCATCAAGAGTAAAGA
	RP: CATAGGTCGGTCCCAACCAAA
Nos2	FP: CTTGTTCAGCTACGCCTTCAACA
	RP: AGAGATTTCTTCAGAGTCTGCCCAT
Rps13	FP: TGCTCCCACCTAATT
	RP: CTTGTGCACACAACAGCAT

## Data Availability

Please contact the corresponding author.

## Supplementary Materials

The following supporting information can be downloaded at: https://www.mdpi.com/article/10.3390/microorganisms10071383/s1. Figure S1: Pre-treatment with UCC118^TM^ does not significantly affect tissue inflammatory markers in a DSS-induced colitis model, Figure S2: Treatment with UCC118^TM^ for 7 days does not alter barrier function but promotes expression of IL-10, Figure S3: UCC118^TM^ treatment promotes M2 marker gene expression during DSS-induced colitis in mice, Figure S4: UCC118^TM^ treatment promotes a healthy faecal microbiome in a DSS-induced colitis model.
