# Supplementary material for: Lactobacillus salivarius UCC118™ Dampens Inflammation and Promotes Microbiota Recovery to Provide Therapeutic Benefit in a DSS-Induced Colitis Model
Source: Microorganisms. 2022 Jul 9;10(7):1383. doi: 10.3390/microorganisms10071383 (PMC9324116; doi:10.3390/microorganisms10071383)
Supplement: Supplementary file 1 [file microorganisms-10-01383-s001.zip › microorganisms-1787995-SI.pdf]

Lactobacillus salivarius UCC118™ dampens inflammation inflammation and promotes microbiota recovery to provide therapeutic benefit in a DSS-induced colitis model

Supplementary Figures

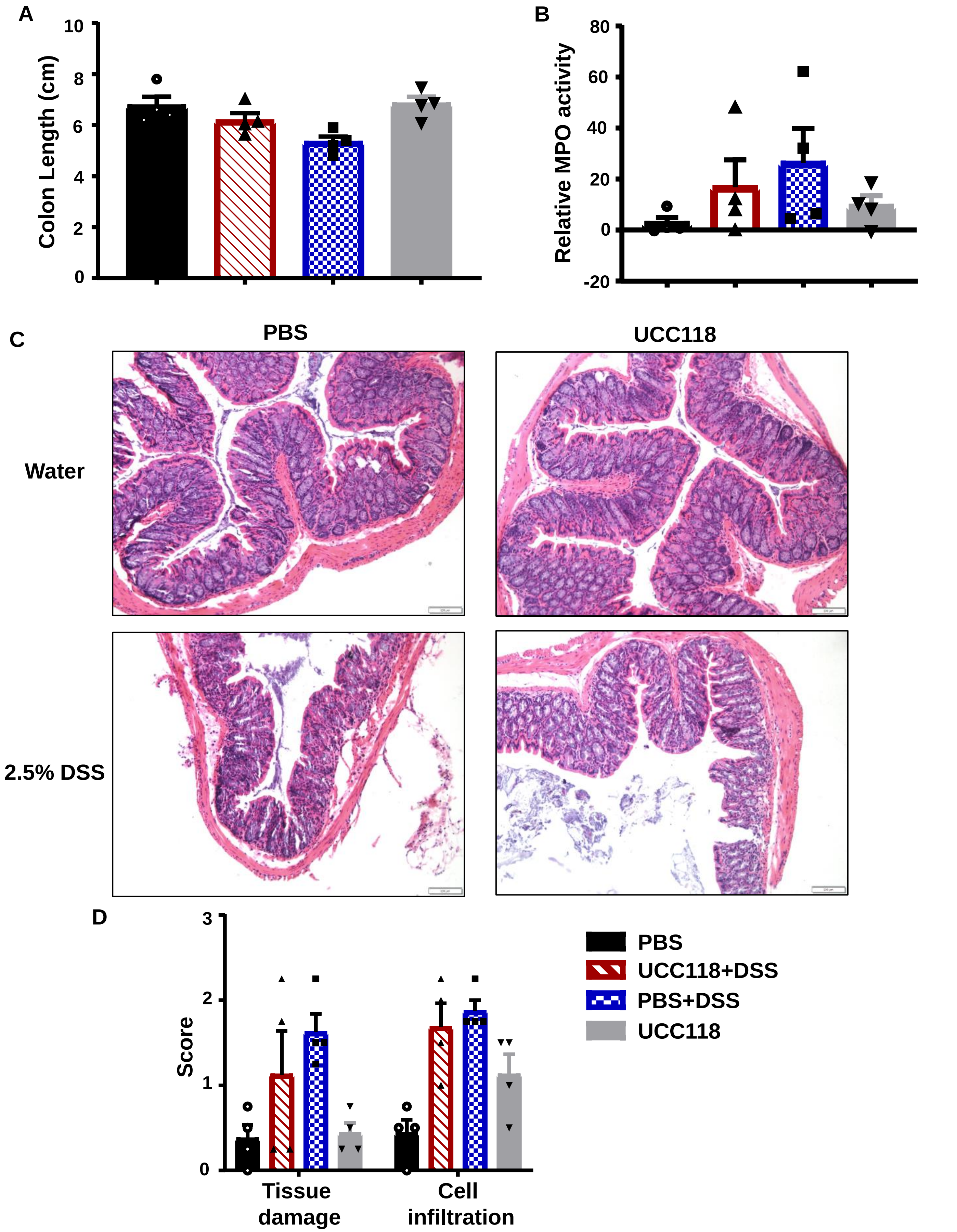

Supplemental Figure S1. Pre-treatment with UCC118™ does not significantly affect tissue inflammatory markers in a DSS-induced colitis model. Colon length (A) and myeloperoxidase activity in colon tissue (B) were quantified. Images of hematoxylin and eosin-stained sections of distal colon tissues from PBS, UCC118™, PBS+DSS and UCC118™+DSS mice (C) were blinded and scored for damage to tissue architecture and cellular infiltration on a scale from 0-3 (D).

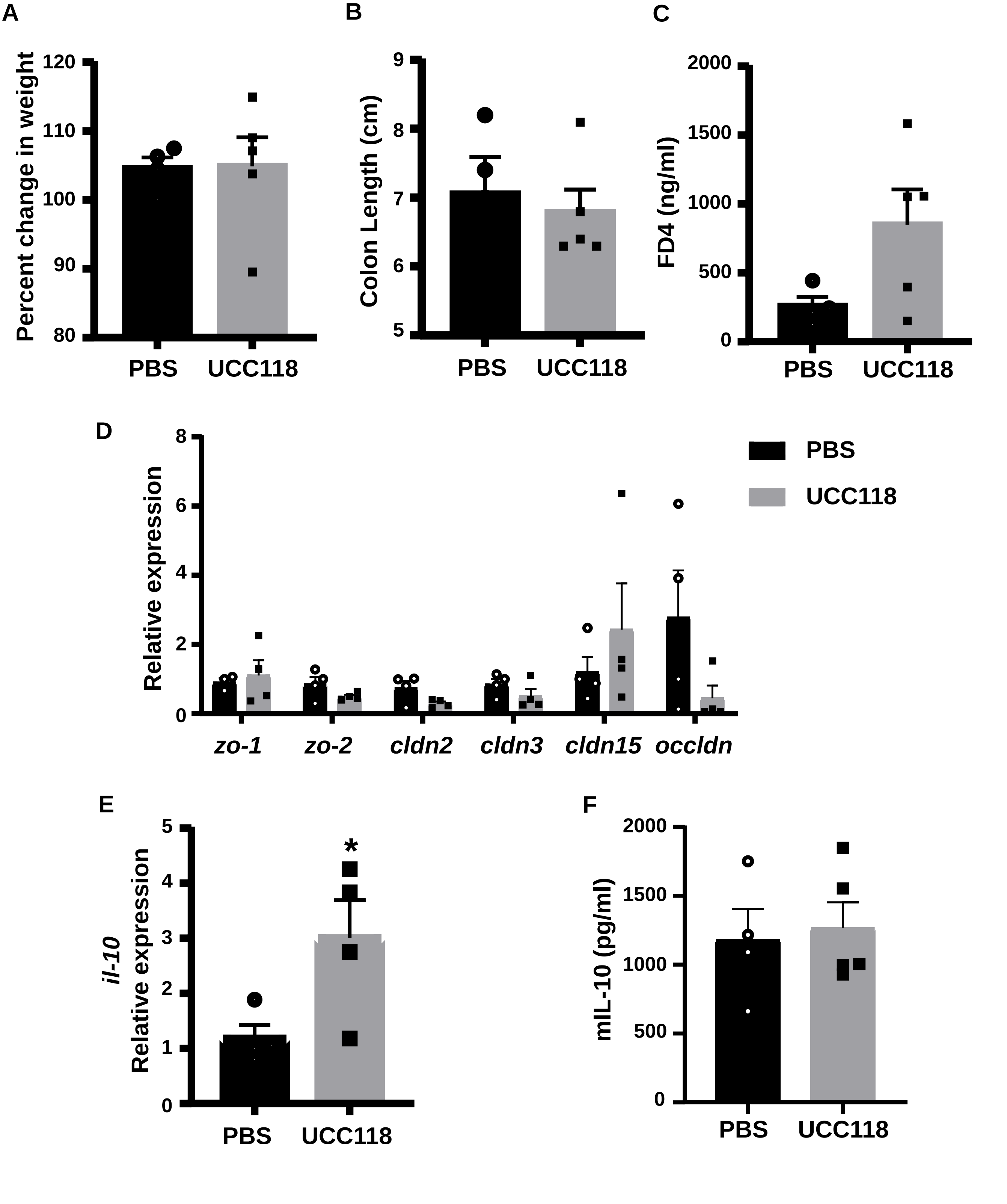

Supplemental Figure S2. Treatment with UCC118™ for 7 days does not alter barrier function but promotes expression of IL-10. C57BL/6JOlaHsd mice were pre-treated with 100µl UCC118™ (10^9 CFU/mouse) or sterile PBS by oral gavage daily for 7 days. Effect of basal UCC118™ treatment on body weight (A), colon length (B), barrier permeability (D) and gene expression of tight junction proteins (D) in the colon were assessed. Colonic IL-10 gene expression (E) and secreted IL-10 (F) were also quantified. n=4 mice in PBS group and n=5 mice in UCC118™ group.

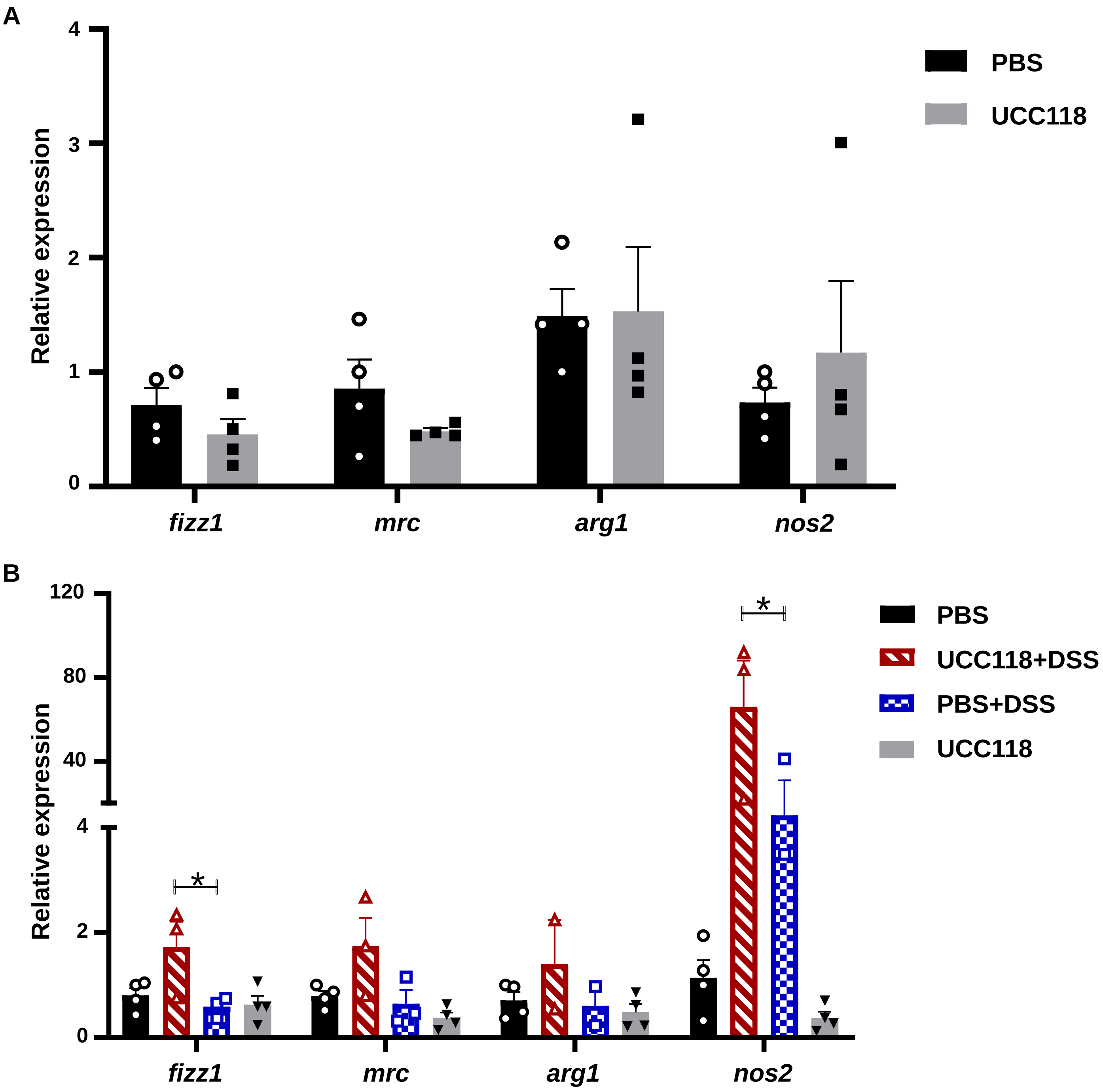

Supplemental Figure S3. UCC118™ treatment promotes M2 marker gene expression during DSS-induced colitis in mice. Gene expression analysis of M1 and M2 markers in distal colon tissues of mice treated with PBS or UCC118™ for 7 days (A). Gene expression analysis of M1 and M2 markers in distal colon tissues of PBS, UCC118™, PBS+DSS and UCC118™ +DSS mice (B). n=4 mice per group. Statistical analysis by one-way ANOVA. *p<0.05.

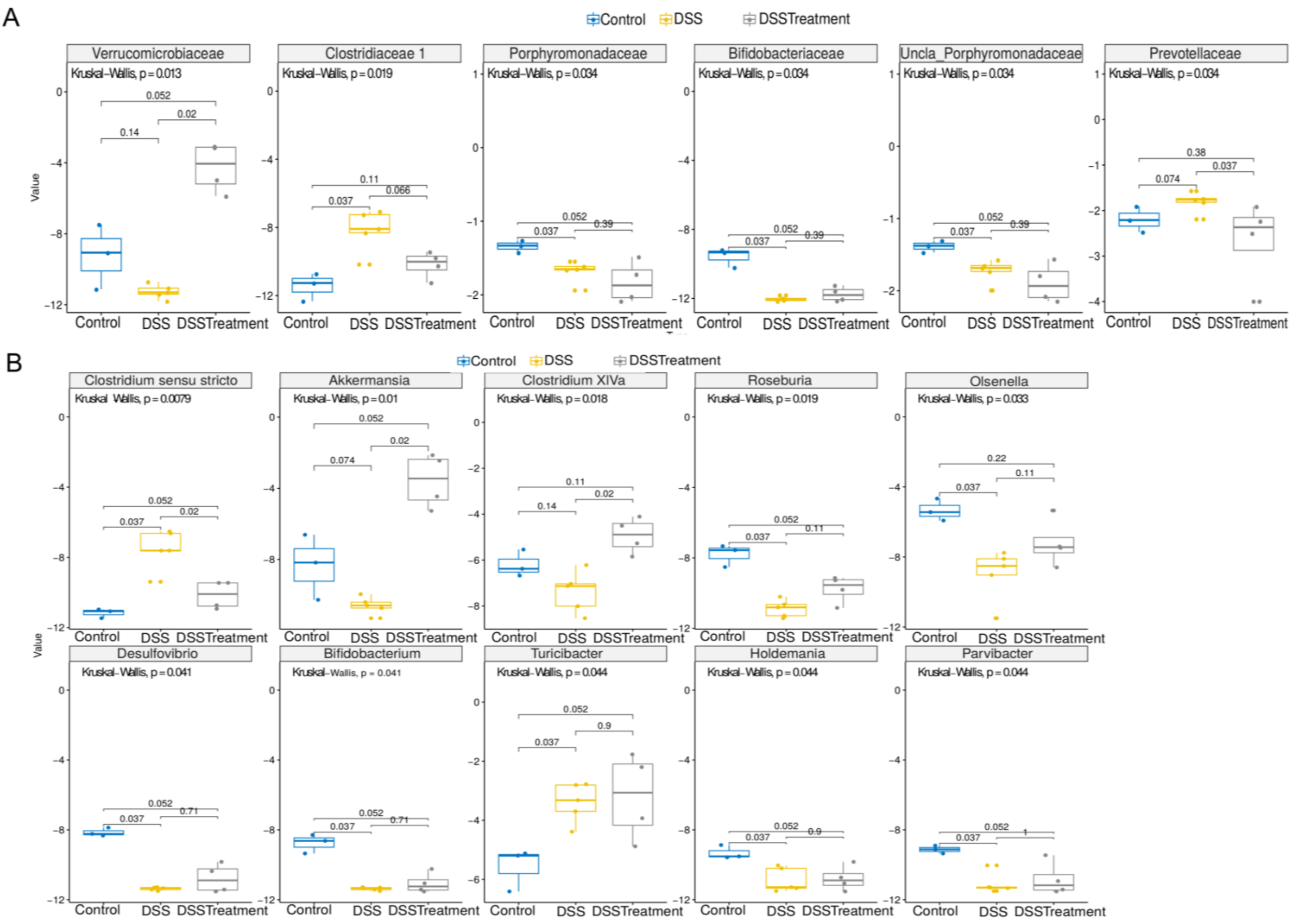

Supplemental Figure S4. UCC118™ treatment promotes a healthy faecal microbiome in a DSS-induced colitis model. The microbiota composition of faecal samples harvested from mice recovering from DSS-colitis with or without UCC118™ treatment (DSS-treatment or DSS-PBS respectively) were analysed by Kruskal-Wallis analysis of differences (A) at family level and (B) at genus level. Results shown are representative of biological groups n≥3, mean values ± SD are presented, Unpaired T-test, * $p<0.05$, ** $p< 0.01$, *** $p<0.001$.
